# Targeted Recombinant Fusion Proteins of IFNγ and Mimetic IFNγ with PDGFβR Bicyclic Peptide Inhibits Liver Fibrogenesis *In Vivo*

**DOI:** 10.1371/journal.pone.0089878

**Published:** 2014-02-24

**Authors:** Ruchi Bansal, Jai Prakash, Marieke De Ruiter, Klaas Poelstra

**Affiliations:** 1 Department of Controlled Drug Delivery (Targeted Therapeutics), MIRA Institute for Biomedical Technology and Technical Medicine, University of Twente, Enschede, The Netherlands; 2 Department of Pharmacokinetics, Toxicology and Targeting, University of Groningen, Groningen, The Netherlands; Centro de Investigación en Medicina Aplicada (CIMA), Spain

## Abstract

Hepatic stellate cells (HSCs), following transdifferentiation to myofibroblasts plays a key role in liver fibrosis. Therefore, attempts to attenuate this myofibroblastic phenotype would be a promising therapeutic approach. Interferon gamma (IFNγ) is a potent anti-fibrotic cytokine, but its pleiotropic receptor expression leading to severe adverse effects has limited its clinical application. Since, activated HSC express high-level of platelet derived growth factor beta receptor (PDGFβR), we investigated the potential of PDGFβR-specific targeting of IFNγ and its signaling peptide that lacks IFNγR binding site (mimetic IFNγ or mimIFNγ) in liver fibrosis. We prepared DNA constructs expressing IFNγ, mimIFNγ or BiPPB (PDGFβR-specific bicyclic peptide)-IFNγ, BiPPB-mimIFNγ fusion proteins. Both chimeric proteins alongwith IFNγ and mimIFNγ were produced in *E.coli*. The expressed proteins were purified and analyzed for PDGFβR-specific binding and *in vitro* effects. Subsequently, these recombinant proteins were investigated for the liver uptake (pSTAT1α signaling pathway), for anti-fibrotic effects and adverse effects (platelet counts) in CCl_4_-induced liver fibrogenesis in mice. The purified HSC-targeted IFNγ and mimIFNγ fusion proteins showed PDGFβR-specific binding and significantly reduced TGFβ-induced collagen-I expression in human HSC (LX2 cells), while mouse IFNγ and mimIFNγ did not show any effect. Conversely, mouse IFNγ and BiPPB-IFNγ induced activation and dose-dependent nitric oxide release in mouse macrophages (express IFNγR while lack PDGFβR), which was not observed with mimIFNγ and BiPPB-mimIFNγ, due to the lack of IFNγR binding sites. *In vivo*, targeted BiPPB-IFNγ and BiPPB-mimIFNγ significantly activated intrahepatic IFNγ-signaling pathway compared to IFNγ and mimIFNγ suggesting increased liver accumulation. Furthermore, the targeted fusion proteins ameliorated liver fibrogenesis in mice by significantly reducing collagen and α-SMA expression and potentiating collagen degradation. IFNγ also induced reduction in fibrogenesis but showed significant decrease in platelet counts, which was restored with targeted proteins. These results suggest that these rationally designed proteins can be further developed as novel anti-fibrotic therapeutics.

## Introduction

IFNγ is a pleiotropic homodimeric Th1 cytokine mainly produced by activated inflammatory cells and has been documented to be highly effective in viral, immunological and malignant diseases [1], [2]. IFNγ (Interferon gamma-1b) has also been explored in clinical trials in patients suffering from liver fibrosis, renal fibrosis or idiopathic pulmonary fibrosis [3]–[6]. However, despite its promising effects *in vivo*, all clinical trials failed due to a lack of efficacy and unfavorable adverse effects [4], [7], [8]. The clinical application of this potent cytokine is nowadays limited to only a very few diseases. Many attempts have been made to prolong the half-life of IFNγ by PEGylation or by increasing its activity through slow release by incorporation in nanoparticles, elastomers, microspheres or liposomes [9]–[11]. These approaches have shown to be beneficial, but adverse effects due to the longer exposure of IFNγ to non-target tissues can still be detrimental. Therefore, targeted approaches leading to an increased therapeutic efficacy without eliciting adverse effects would be ideal to treat slowly progressing chronic diseases [12].

Liver fibrosis, induced by viral infections (e.g hepatitis B and C), alcohol abuse, metabolic syndrome or genetic disorders, is characterized by an excessive accumulation of matrix proteins in the liver [13], [14]. Worldwide millions of people, suffering from one of these disorders, are at risk of developing liver fibrosis. Currently, there are no effective and clinically approved anti-fibrotic therapy available and the treatment is mainly based on the removal of the underlying cause of the disease [15], [16]. Liver transplantation is the only option for the patients suffering from advanced liver fibrosis or end stage liver cirrhosis.

Hepatic stellate cells (HSC) are the key pathogenic cells involved in the progression of liver fibrosis. These cells gets activated following release of growth factors from damaged hepatocytes, kupffer cells and infiltrating inflammatory cells. These activated HSC are then transformed into proliferative and contractile myofibroblast-like cells that produce large amounts of extracellular matrix (ECM) proteins leading to impairment of the structure and function of liver [17], [18]. Among the potent anti-fibrotic therapeutic cytokines, Interferon gamma (IFNγ) is shown to be highly efficacious *in vitro* and *in vivo* in liver fibrosis models [19], but it failed in clinical trials due to reduced efficacy and unwanted systemic effects [8].

Others and we have shown that activated HSC abundantly express the platelet derived growth factor receptor (PDGFβR) during liver fibrosis, while its expression is relatively weak on other cells and normal tissues [20]–[22]. Recently, we have shown that using PDGFβR-specific delivery of IFNγ to activated HSC; acute and advanced liver fibrosis *in vivo* could be significantly inhibited with minimal adverse effects [20], [23]. The results of these chimerical constructs of IFNγ and PDGFβR binding moieties were remarkably potent and encouraged us to pursue this strategy and prepare a targeted fusion proteins that are inexpensive and can be feasibly applied in clinical trials.

To that end, we have now produced the recombinant proteins containing a bicyclic PDGFβR-recognizing peptide (BiPPB) fused to IFNγ to synthesize BiPPB-IFNγ or to the signaling moiety of IFNγ (mimetic IFNγ or mimIFNγ) lacking extracellular IFNγR binding site [24], [25] to generate BiPPB-mimIFNγ in *E.coli*. IFNγ and mimetic IFNγ were also expressed in parallel as control proteins. These proteins were analyzed *in vitro* in human HSC cells and *in vivo* in acute liver fibrogenesis mouse model. Encouragingly, we found that the targeted fusion proteins (BiPPB-IFNγ and BiPPB-mimIFNγ) specifically bound to PDGFβR-expressing human HSC and induced significant reduction in major ECM production *in vitro* (collagen). *In vivo*, in the CCl_4_ mouse model, targeted proteins significantly stimulated the IFNγ-mediated pSTAT1α-signaling pathway, inhibited collagen accumulation, HSC activation and induced fibrolysis. Apart from therapeutic effects, BiPPB-mimIFNγ and to a lesser extent BiPPB-IFNγ did not affect circulating platelets counts as observed by untargeted IFNγ.

## Materials and Methods

### Plasmids, Bacterial Strains and Cell Culture

pET42a and pET39b protein expression vectors were purchased from Novagen (Madison, WI, USA). All the restriction enzymes were procured from New England Biolabs (Beverly, MA). *Escherichia coli* strain JM109 was used for plasmid propagation and cloning. Strain BL21 (DE3) (Novagen) was used as a host for the production of recombinant proteins. Human hepatic stellate cells, LX2 were kindly provided by Prof. Scott Friedman (Mount Sinai Hospital, New York). LX2 cell line is a well-established human HSC cell line [26]. LX2 cells were cultured in DMEM-Glutamax (Invitrogen, Carlsbad, CA) supplemented with 10% FBS and antibiotics (50 U/ml penicillin and 50 ng/ml streptomycin). Mouse spleen cells, freshly isolated from healthy C57BL/6 mice, were grown in DMEM cell culture medium.

### Plasmid Construction

#### (a) Preparation of pET42a-IFNγ and pET42a-mimIFNγ

Splenocytes (freshly isolated from the spleen of healthy C57BL/6 mice) were seeded in the presence of 20 µg/ml phytohemagglutinin (PHA) for 24 h. RNA was isolated from spleen cells was used for PCR amplification of mouse IFNγ and mimetic IFNγ using gene specific primers (primer 1, primer 2 for IFNγ and primer 3, primer 4 for mimIFNγ as listed in **Table 1**). The PCR product was purified and digested with Eco RI. The digested gene was inserted into Psh A1/Eco RI digested pET42a vector to produce pET42a-IFNγ or pET42a-mim IFNγ (**Figure S1 and S2**).

**Table 1:** Primers used for plasmids construction.

Primers	Sequence	Restrictionsites
**Primer 1**	TGCCACGGCCACAGTCATTGAAAGC	–
**Primer 2**	GCGAATTCTCAGCAGCGACTCCTTTTC	Eco RI
**Primer 3**	GCCAAGTTTGAGGTCAACAACCCACAG	–
**Primer 4**	GCGAATTCTCAGCGACTCCTTTTCCG	Eco RI
**Primer 5**	TGTTCTAGAAACCTCATCGATTGTAAG	–
**Primer 6**	GCGGATCCCTTACAATCGATGAGGTT	Bam HI
**Primer 7**	GCGGATCCGGAGGTTGTTCACGTAATCTAATAG	Bam HI
**Primer 8**	TAGCGGCCGCTGAACAATCTATTAGATTA	Not I
**Primer 9**	GGGCGGCCGCATGTCATGGTACAGTCATTGAA	Not I
**Primer 10**	GCCTCGAGTTAGCAGCGACTCCTTTTCCG	Xho I
**Primer 11**	AAGCGGCCGCAGCCAAGTTTGAGGTCAACAAC	Not I
**Primer 12**	GCCTCGAGTTATCGACTCCTTTTCCGCTTCCTG	Xho I

#### (b) Preparation of pET39b-BiPPB-IFNγ and pET39b-BiPPB-mimIFNγ

For synthesis of pET39b-BiPPB-IFNγ and pET39b-BiPPB-mimIFNγ, pET39b-BiPPB was prepared by PCR extension of annealed primers (primer 5, primer 6 and primer 7, primer 8 listed in **Table 1**). The resulting products (2 fragments) were then digested with Bam HI and Not I, purified and ligated to Sca I/Not I digested pET39b vector (**Figure S3**). Thereafter, PCR amplified IFNγ and mimIFNγ (primer 9, primer 10 for IFNγ and primer 11, primer 12 for mimIFNγ listed in **Table 1**) were digested with Not I/Xho I and inserted into Not I/Xho I digested and purified pET39b vector. The resulted recombinant vectors were termed as pET39b-BiPPB-IFNγ and pET39b-BiPPB-mimIFNγ that encodes for BiPPB and IFNγ or mimIFNγ plus a three amino-acid linker (AAA) between them (**Figure S4 and S5**).

All the DNA constructs were confirmed by DNA sequencing. pET42a-IFNγ and pET42a-mimIFNγ expressed proteins of 45.2 and 34.5 KDa respectively including GST tag, His tag, S tag and IFNγ or mimIFNγ. pET39b-BiPPB-IFNγ and pET39b-BiPPB-mimIFNγ expressed 42.8 and 32.2 KDa fusion proteins respectively including Dsb tag, His tag, BiPPB, linker and IFNγ or mimIFNγ.

### Expression and Purification of Recombinant Proteins

DNA constructs were transformed into CaCl_2_ competent BL21 (DE3) and then induced with 1 mM IPTG at 37°C for 4 h.

For purification of IFNγ and mimIFNγ, the cell pellets were washed and suspended in binding buffer (500 mM NaCl, 20 mM sodium phosphate buffer, 20 mM Imidazole pH7.4), followed by enzymatic lysis (0.2 mg/ml lysozyme, 20 µg/ml DNase, 10 mM PMSF) for 30 min at 4°C and sonication for 10 min (10–15 short bursts). The cell debris was removed by centrifugation at 12,000 g for 30 min at 4°C. The supernatant was extensively dialyzed against binding buffer.

For purification of periplasmic proteins BiPPB-IFNγ and BiPPB-mimIFNγ, the cell pellets were suspended in 0.03 mol/l Tris.HCl, 20% sucrose, 0.001 mol/l EDTA, pH8.0. The cells were incubated on ice for 5–10 min followed by centrifugation at 8000 g for 20 min at 4°C. The pellet was then resuspended in ice-cold 5 mM MgSO_4_ and stirred for 10 min on ice bath. The cell debris was removed by centrifugation at 8000 g for 20 min at 4°C. The supernatant was extensively dialyzed against binding buffer.

The supernatants were then applied to Ni-charged chelating-Sepharose HisTrap High performance column (Pharmacia Biotech, Uppsala, Sweden). After being washed with binding buffer, the protein was eluted with the elution buffer containing 500 mM imidazole and then dialysed against PBS buffer overnight.

### Dot Blot Immunoassay

Purified proteins were applied on dehydrated PVDF membrane (Roche, Mannheim, Germany) in dot-blot apparatus (Bio-Rad, Hercules, CA, USA). The wells were then incubated with 200 µl blocking solution (1% BSA in TBS) for 1 h. After washing in TBST (0.05% tween 20 in TBS), the membrane was incubated for 1 h with either anti-IFNγ antibody (1∶2000; Abcam, Cambridge, UK) or anti-PPB antibody (1∶1000, custom-made, Harlan) followed by 1 h incubation with horseradish peroxidase-labeled secondary antibody. The membrane was washed again with TBST subsequent with incubation in substrate solution (0.06% diaminobenzidine (DAB), 0.08% hydrogen peroxide in TBS) for color development. The membranes were finally rinsed in distilled water and air-dried.

### In vitro Binding of Recombinant Proteins in Human HSC (LX2 Cells)

Cells (30,000 cells/well) were seeded in 24-well culture plates (Becton Dickinson, Heidelberg, Germany) and grown overnight at 37°C/5%CO_2_. Then, cells were incubated with IFNγ, mimIFNγ, BiPPB-IFNγ or BiPPB-mimIFNγ (1 µg/ml) for 2 h. Thereafter, cells were fixed and stained using anti-PPB antibody.

### In vitro Effects of Recombinant Proteins in Human HSC

Cells were plated in 24 well (30,000 cells/well) and 12 well (75,000 cells/well) culture plates, grown overnight and were starved with 0.5% containing medium for 24 h. Starved cells were then incubated with medium alone, recombinant IFNγ, mimIFNγ, BiPPB-IFNγ or BiPPB-mimIFNγ (equivalent to 1 µg/ml) plus 5 ng/ml of human recombinant TGFβ1 (Roche) for 48 h. Subsequently, cells (24 well plates) were fixed and stained for collagen I (1∶100, Southern Biotech, Birmingham, AL). In addition, cells (12 well plates) were lysed with 5x SDS-PAGE sample buffer constituted with β-mercaptoethanol (Stratagene, La Jolla, CA) to perform western blot analysis for collagen I (1∶250; Southern Biotech) and β-actin (house-keeping gene) as per standard protocols.

### Nitric Oxide (NO) Release Bioassay in Mouse RAW Macrophages

The biological activity of recombinant proteins were assessed by measuring the accumulation of nitrite NO2, a stable nitric oxide (NO) metabolite produced by RAW macrophages as described previously [27]. Briefly, cells (1×10^5^cells/200 µl/well) were seeded in 96-well plates and grown overnight at 37°C/5%CO2. Then cells were incubated with either medium alone or recombinant proteins at different concentrations (10, 25, 50, 100 and 200 ng/ml) together with 100 ng/ml lipopolysaccharides (LPS from E. coli 055:B5, Sigma). After 24 h, the secreted nitrite was measured using Griess reagent (1% sulfanilamide; 0.1% naphthylethylendiamine dihydrochloride; 3% H3PO4). The absorbance was determined at 550 nm using an ELISA plate reader.

### Animal Experiments: Ethics Statement

All the animal experiments were performed in strict accordance with the guidelines and regulations for the Care and Use of Laboratory Animals, University of Groningen, The Netherlands. The protocols were approved by the Institutional Animal Ethics Committee of the University of Groningen, The Netherlands (Permit Number 5429A). Male 6- to 8-week old C57BL/6 mice were purchased from Harlan (Zeist, Netherlands) and kept at 12 h light/12 h dark cycles with ad libitum normal diet.

### CCl_4_-induced Acute Liver Fibrogenesis Model in Mice

Acute liver injury was induced in male C57BL/6 mice by a single intra-peritoneal injection of carbon tetrachloride (CCl_4_; 1 ml/kg prepared in olive oil) at day 1. At day 2 and day 3, mice received intravenous injections of IFNγ (n = 6), mimIFNγ (n = 5), BiPPB-IFNγ (n = 6), BiPPB-mimIFNγ (n = 6) (equivalent to 5 µg IFNγ/mouse/day) or PBS alone. At day 4, all mice were sacrificed under deep anesthesia by cervical dislocation; blood and livers were collected for subsequent measurements.

### In vivo IFNγ (pSTAT1α) Signaling Pathway

The IFNγ signaling pathway was analyzed in the acute liver fibrogenesis model in mice after 24 h of administration with IFNγ (n = 6), mimIFNγ (n = 5), BiPPB-IFNγ (n = 6), BiPPB-mimIFNγ (n = 6) (equivalent to 5 µg IFNγ/mouse/day) or PBS alone. Liver tissues samples were homogenized in cold RIPA buffer (50 mM Tris-HCl, 150 mM NaCl, 0.1% SDS, 0.1% Igepal in 0.5% sodium deoxycholate, 1 tablet of protease inhibitor cocktail and 1 tablet of phosphatase inhibitor in 10 ml) on ice using a tissue homogenizer and the lysates were centrifuged at 12,000 rpm for 1 h at 4°C. 20 µg of protein was used for western blot analysis performed as per standard protocols using anti-pSTAT1α antibody (1∶1000; cell signaling, Beverly, MA) or anti-β-actin antibody (1∶5000; Sigma).

### Immunohistochemistry and Immunofluorescence

The cells were fixed with acetone:methanol (1∶1), dried and stored until immunostaining. Livers were harvested and transferred to Tissue-Tek OCT embedding medium, and snap frozen in isopentane chilled in a dry ice. Cryosections (4 µm) were cut using a Leica CM 3050 cryostat (Leica Microsystems, Nussloch, Germany). The sections were allowed to adhere to Superfrost microscopic glass slides (Menzel-Gläser, Braunschweig, Germany), air-dried and fixed with acetone for 10 min. Cells or tissue sections were rehydrated with PBS and incubated with the collagen I antibody (1∶100; Southern Biotech), α-SMA antibody (1∶600, Sigma) or PPB (1∶100; custom-made, Harlan) for 1 h. Thereafter, cells or sections were washed thrice with PBS and incubated with horseradish peroxidase (HRP)-conjugated secondary antibody for 30 min. Cells or sections were washed again and further incubated with HRP-conjugated tertiary antibody for 30 min. Thereafter, peroxidase activity was developed with 3-amino-9-ethyl carbazole (Sigma, St. Louis,MO) for 20 min and nuclei were counterstained with hematoxylin (Fluka Chemie, Buchs, Switzerland). Cells or sections were then mounted with Kaiser’s gelatin (Darmstadt, Germany), visualized and photographed using a light microscope (Olympus UK Ltd., Essex, UK).

### Quantitative Real-time PCR

Total RNA from liver tissues was isolated using RNeasy mini kit (Qiagen, Hilden, Germany) according to manufacturer’s instructions. RNA concentrations were quantitated by a Nanodrop UV spectrophotometer (NanoDrop Technologies, Wilmington, DE). Total RNA (1.6 µg) was reverse transcribed in a volume of 50 µl using cDNA synthesis kit (Promega, Madison, WI). All primers were purchased from Sigma-Genosys (Haverhill, UK). Following primers were used, MMP13 forward: CCAGAACTTCCCAACCATGT; MMP13 reverse: GTCTTCCCCGTGTTCTCAAA; TIMP1 forward: ATCAGTGCCTGCAGCTTCTT; TIMP1 reverse: TGACGGCTCTGGTAGTCCTC; GAPDH forward: ACAGTCCATGCCATCACTGC; GAPDH reverse: GATCCACGACGGACACATTG. The reactions were performed with 20 ng cDNA using SYBR green PCR master mix (Applied Biosystems, Foster City, CA) according to manufacturer’s instructions and were analyzed by ABI7900HT sequence detection system (Applied Biosystems). Finally, the threshold cycles (Ct) were calculated and relative gene expression was normalized with GAPDH (for mouse) as housekeeping gene.

### Statistical Analyses

Data are presented as mean ± standard error mean (SEM). Multiple comparisons between different groups were performed by one-way ANOVA with Bonferroni post-test using GraphPad Prism version 5.02 (GraphPad Prism Software, Inc., La Jolla, CA, USA).

## Results

### Construction and Expression of Recombinant Proteins

**Figure 1:**
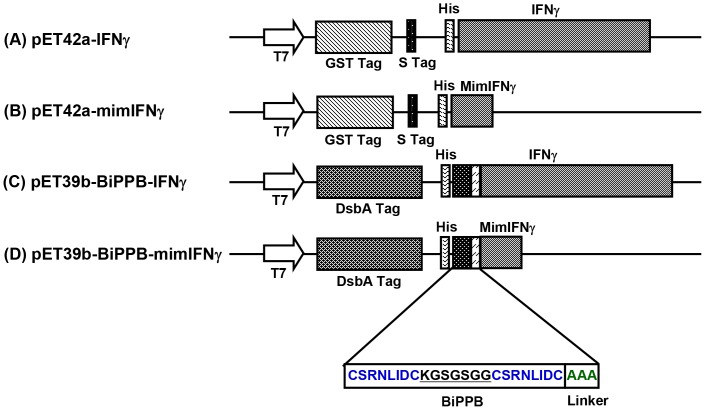
Schematic representation of the prokaryotic vectors used for the expression of the recombinant proteins. IFNγ (**A**) and mimetic IFNγ (**B**) were cloned in-frame upstream of His-tag in pET42a (+) vector to achieve cytoplasmic protein expression. The fusion proteins BiPPB-IFNγ (**C**) and BiPPB-mimIFNγ (**D**) were expressed in pET39b (+) vector for periplasmic expression of fusion proteins to ensure proper folding and disulfide bonds formation. For the synthesis of fusion proteins, BiPPB was fused to the N-terminal of IFNγ or mimetic IFNγ sequence through a flexible 3 amino acid linker (AAA) maintaining the open reading frame.

**Figure 2:**
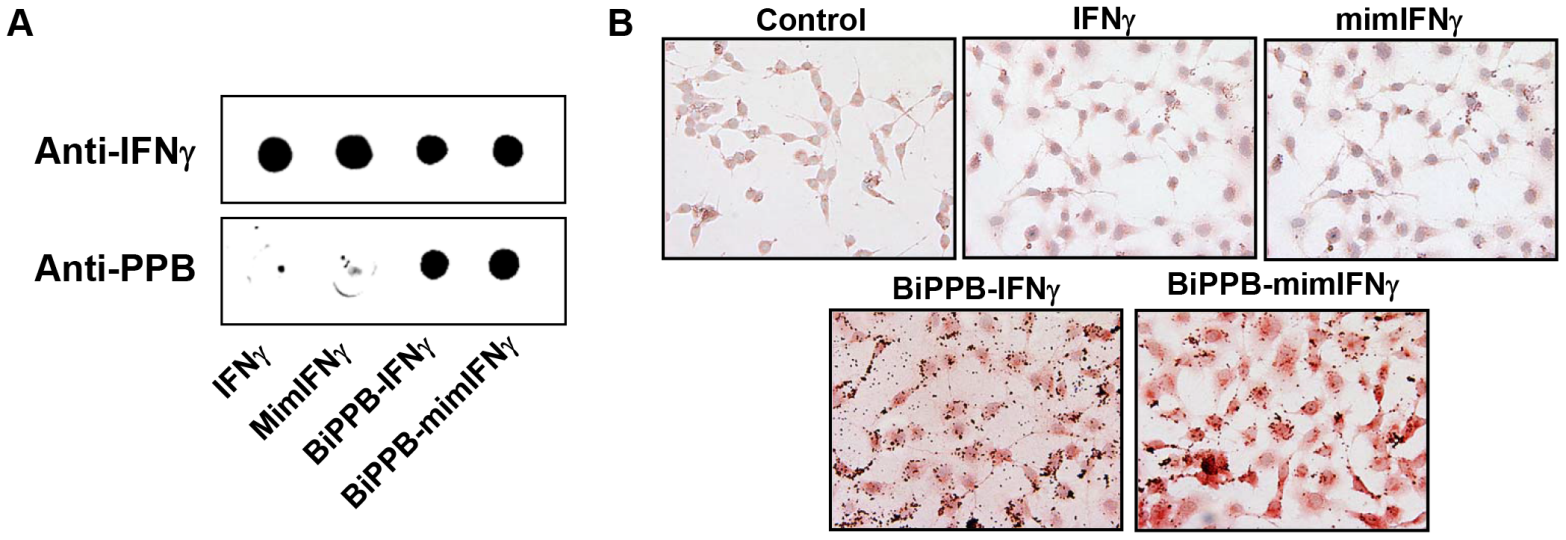
Dot-blot and *in vitro* binding of recombinant proteins in human HSC. (**A**) Dot-blot analysis of purified recombinant proteins IFNγ, mimetic IFNγ, BiPPB-IFNγ and BiPPB-mimIFNγ using anti-IFNγ and anti-PPB antibody. (**B**) Representative pictures showing binding of BiPPB-IFNγ and BiPPB-mimIFNγ to human HSC (LX2). Mouse IFNγ and mimIFNγ did not show any binding (similar to control) to human LX2 cells due to species differences and lack of IFNγR or PDGFβR binding sites respectively.

We synthesized four recombinant proteins: IFNγ, mimetic IFNγ (mimIFNγ), BiPPB-IFNγ and BiPPB-mimetic IFNγ (BiPPB-mimIFNγ) (**Figure 1**). IFNγ and mimetic IFNγ were expressed using pET42a derived bacterial expression vector to achieve cytoplasmic expression of the proteins (**Figure 1A, 1B** **and Figure S1, S2**). While, BiPPB-IFNγ and BiPPB-mimIFNγ fusion proteins were expressed using pET39b (+) expression vector (**Figure 1C, 1D** **and Figure S4, S5**) to yield periplasmic expression, required for their proper folding. DNA constructs were extensively analysed for the presence of inserts using specific restriction enzyme digestions and by automated DNA sequencing. Thereafter, constructs were transformed into *E. coli* strain BL21 (DE3) and protein expression was induced by 1 mM IPTG. Proteins of interest with the expected molecular weight were found in total cell pellets. Soluble proteins were purified from the supernatant of lysed bacterial pellets through Ni-NTA affinity columns. Purified proteins were further dialysed against PBS and concentrated by ultrafiltration. The presence of IFNγ moieties or/and PPB peptides in the prepared fusion proteins was confirmed in dot blots using anti-IFNγ and anti-PPB antibodies (**Figure 2A**).

### In vitro Binding of Recombinant Proteins in Human HSC

The binding of IFNγ to its receptor is strictly species-specific, whereas PDGFβR binding is not. In order to demonstrate PDGFβR-specific binding of BiPPB containing fusion proteins, we used human LX2 hepatic stellate cells that are known to express PDGFβR. The results confirmed the species specificity of IFNγ; mouse IFNγ and mimetic IFNγ (lacking extracellular IFNγR binding site) did not show any binding to human HSC (**Figure 2B**). However, BiPPB-fused mouse IFNγ or mimetic IFNγ proteins showed high binding to human cells (**Figure 2B**).

### In vitro Effects of Recombinant Proteins in Human HSC and Mouse Macrophages

**Figure 3:**
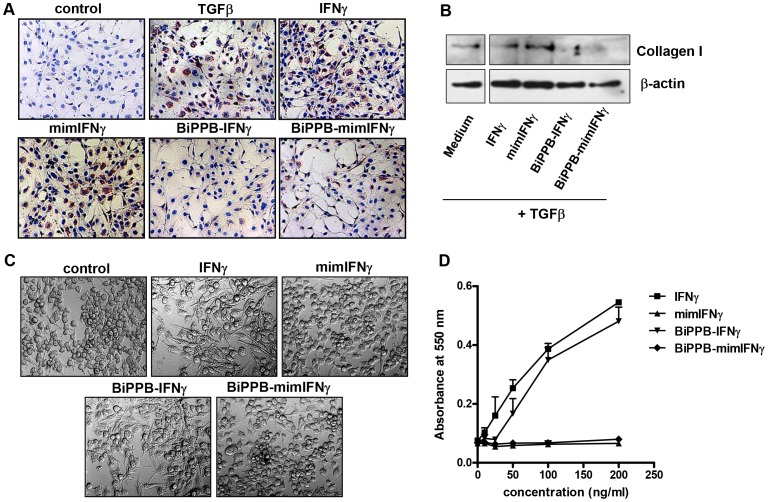
*In vitro* effects of the recombinant proteins in human HSC and mouse macrophages. Representative pictures (**A**) and western blot analysis (**B**) of collagen-I stained LX2 cells, incubated with TGFβ (5 ng/ml) in combination with different recombinant proteins (1 µg/ml). In human LX2 cells, only BiPPB-modified proteins attenuated collagen expression, whereas unmodified mouse IFNγ and mimIFNγ did not cause any reduction due to species restriction and lack of receptor binding sites respectively. (**C**) Representative microscopic photographs depicting the activation of mouse RAW macrophages after 24 h of incubation with mouse IFNγ and BiPPB-IFNγ (1 µg/ml) along with 100 ng/ml LPS. (**D**) Dose-dependent release of nitrogen oxide (NOx) in mouse RAW macrophages after incubation with unmodified IFNγ and BiPPB modified IFNγ fusion protein. MimIFNγ and BiPPB-mimIFNγ did not induce any NOx release due to absence of IFNγR binding site and/or lack of PDGFβR on RAW macrophages.

Following the binding studies, we investigated the anti-fibrotic effects of the recombinant proteins in human HSC after their activation with TGFβ. In corroboration with the binding studies in human LX2 cells, TGFβ-induced collagen expression was strongly inhibited by treatment with the PDGFR-specific BiPPB-IFNγ and -mimIFNγ fusion proteins (**Figure 3A, 3B**) as analyzed by immunostaining and western blot analysis. While both mouse IFNγ and mimIFNγ did not induce any effect in human cells due to species differences and the absence of a receptor-binding site respectively. On the other hand, mouse IFNγ and BiPPB-IFNγ strongly potentiated LPS-induced activation and dose-dependent release of nitric oxide in mouse RAW macrophages due to presence of mouse IFNγR (**Figure 3C, 3D**). However, mimIFNγ lacking the IFNγR binding site and mimIFNγ-BiPPB directed to PDGFβR did not induce any effect due to lack of PDGFβR expression on macrophages (**Figure 3C, 3D**).

### In vivo Liver Uptake of BiPPB-modified Fusion Proteins in Acute CCl_4_-induced Liver Fibrosis in Mice

**Figure 4:**
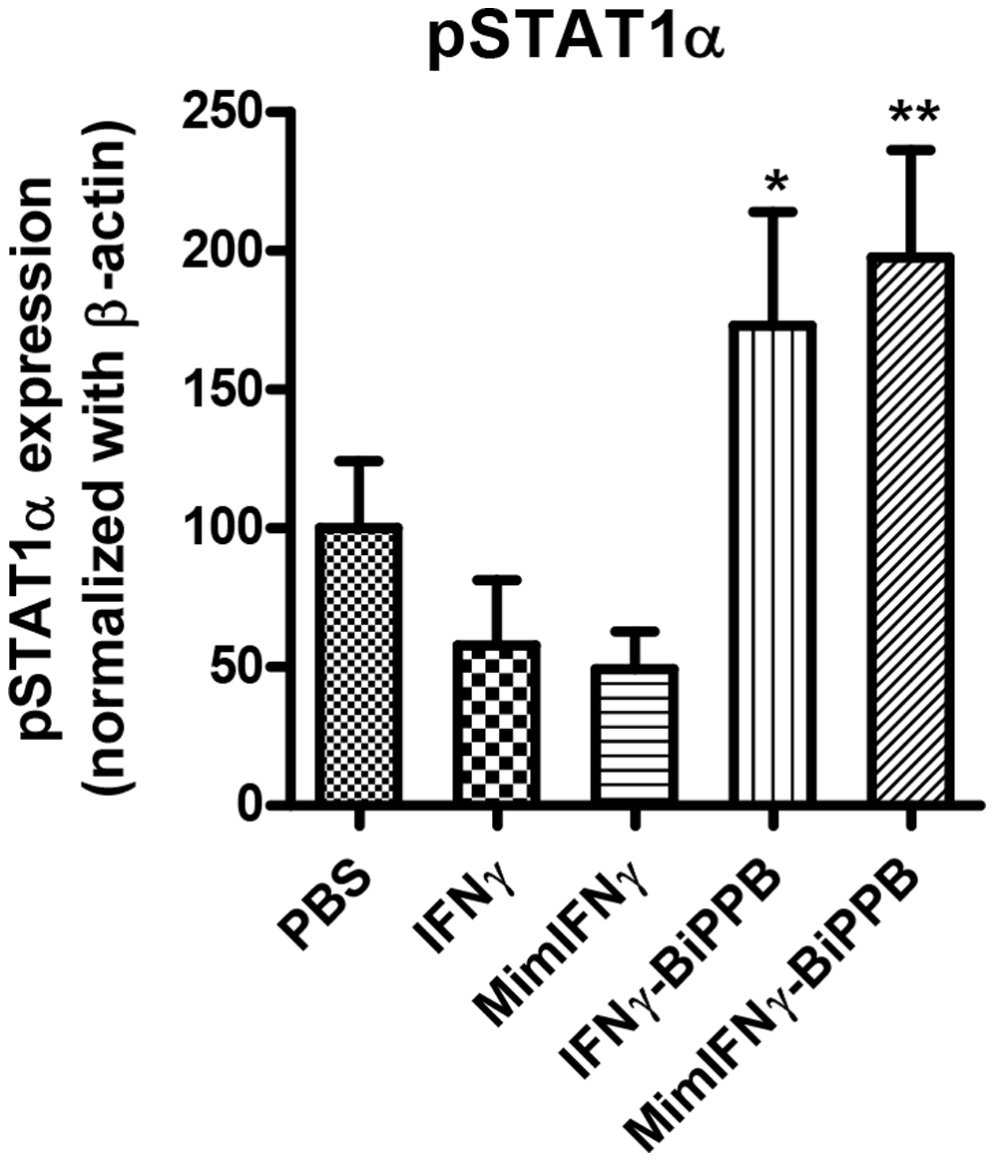
*In vivo* activation of IFNγ pSTAT1α signaling pathway by recombinant proteins. Western blot analysis of pSTAT1α after 24 h post intravenous administration of IFNγ (n = 6), MimIFNγ (n = 5), BiPPB-IFNγ (n = 6), BiPPB-mimIFNγ (n = 6) or PBS alone (n = 6). The graph shows significant increase in activation of pSTAT1α with HSC-targeted fusion proteins (BiPPB-IFNγ, BiPPB-mimIFNγ). Bars represent mean ± SEM of 5–6 mice per group. *P<0.05 and **P<0.01 versus PBS treated CCl_4_ mice. The groups were normalized to CCl_4_ group (treated with PBS).

Others and we have earlier demonstrated that PDGFβR is highly upregulated on activated HSC during acute and advanced liver fibrosis [20], [21]. Our group has extensively studied the bio-distribution of proteins modified with PDGFβR-recognizing cyclic peptides (PPB), using radiolabeled and imaging studies, and showed high distribution of PPB-modified proteins to PDGFβR-expressing hepatic stellate cells in fibrotic livers [23]. In the present study, we assessed the intracellular activation of the pSTAT1α signaling pathway after 24 h of IFNγ, mimIFNγ, BiPPB-IFNγ and BiPPB-mimIFNγ administration in CCl_4_-induced liver fibrosis in mice. We found a significant increase in pSTAT1α activation after 24 h of treatment with BiPPB-IFNγ (p<0.05) and BiPPB-mimIFNγ (p<0.01) compared to PBS, IFNγ and mimIFNγ treatments indicating enhanced liver uptake of the fusion proteins due to high PDGFβR expression on activated HSC in the fibrotic livers (**Figure 4** **and Figure S6**).

### In vivo Effects of BiPPB-modified Fusion Proteins in Acute CCl_4_-induced Liver Fibrosis Model in Mice

**Figure 5:**
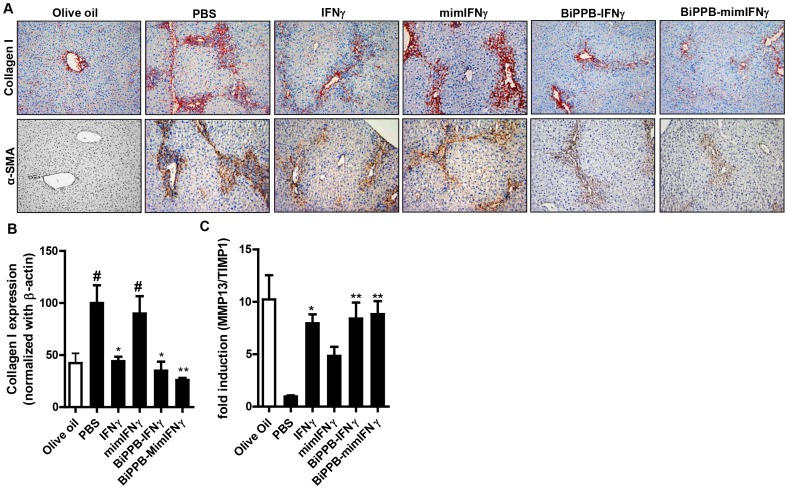
Effects of recombinant proteins on fibrotic parameters in vivo. (**A**) Representative pictures of collagen I and α-SMA stained liver sections from olive oil treated mice (normal) or CCl_4_-treated mice (acute model) that were treated with IFNγ (n = 6), MimIFNγ (n = 5), BiPPB-IFNγ (n = 6), BiPPB-mimIFNγ (n = 6) or PBS alone (n = 6). Scale bars, 200 µm. (**B**) Whole-liver lysates from treated animals were subjected to western blot analysis using anti-collagen I antibody. Graph represents collagen I expression (normalized with β-actin) depicted as mean ± SEM from n = 5–6 mice per group. #P<0.05 denotes significance versus PBS treated olive oil mice and *P<0.05, **P<0.01 denotes significance versus PBS treated-CCl_4_ mice. For quantitative analysis, the groups were normalized to vehicle group (PBS treated-CCl_4_ mice). (**C**) Effect of recombinant proteins on intrahepatic fibrinolysis as determined by the ratio of MMP13 and TIMP-1 transcripts. The groups were normalized to vehicle group (PBS treated-CCl_4_ mice). Bars represent mean ± SEM of 5–6 mice per group. *P<0.05, **P<0.01 denotes significance versus PBS treated-CCl_4_ mice.

A single CCl_4_ administration induced acute liver injury in mice, characterized by an increased intrahepatic expression of HSC activation marker (α-smooth muscle actin) and an increased deposition of extracellular matrix molecule (collagen I) as shown in **Figure 5A–5C**. IFNγ, mimIFNγ, BiPPB-IFNγ and BiPPB-mimIFNγ proteins were examined for their anti-fibrotic effects in this experimental model in mice. After two intravenous injections, a strong reduction in collagen I expression was observed with IFNγ (≈45% reduction, p<0.05), HSC-targeted BiPPB-IFNγ (≈50% reduction, p<0.05) and BiPPB-mimIFNγ (≈60% reduction, p<0.01) as analyzed by immunostaining and quantitative western blot analysis (**Figure 5A, 5B** **and Figure S7)**. Furthermore, α-SMA expression was attenuated by HSC-targeted BiPPB-IFNγ administration and more strongly by BiPPB-mimIFNγ treatment as shown in **Figure 5A** **,** while IFNγ had only little effect and mimIFNγ did not induce any reduction in α-SMA expression levels **(** **Figure 5A** **)**. Apart from collagen expression and deposition, the balance between collagen degrading matrix metalloproteinases-13 (MMP-13) and their major endogenous inhibitor, tissue inhibitor of metalloproteinases-1 (TIMP-1), is an important determinant of progression or reversal of fibrosis [28], [29]. A significant increase in MMP13/TIMP1 transcript ratio was observed after treatment with IFNγ (p<0.05), BiPPB-IFNγ (p<0.01) and BiPPB-mimIFNγ (p<0.01) suggesting activation of fibrolysis and induction of reversal of fibrosis (**Figure 5C**). Of note, no effect was observed with mimetic IFNγ alone due to lack of an IFNγR binding site while the inhibitory effects of HSC-targeted BiPPB-modified fusion proteins on collagen deposition were higher compared to non-targeted IFNγ (**Figure 5**).

**Figure 6:**
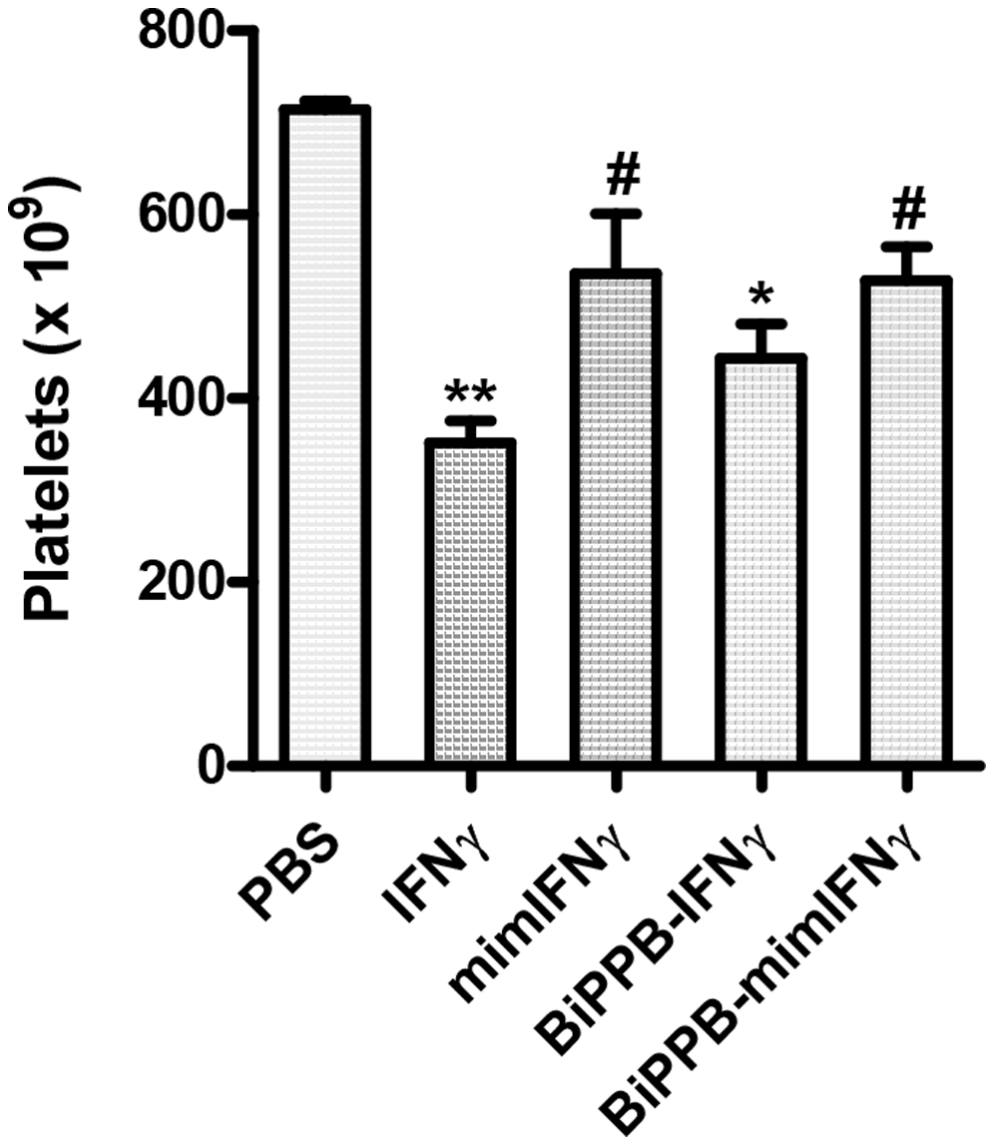
Effects of recombinant proteins on IFNγ-related adverse effect in the acute CCl_4_ model. Graph represents platelet counts measured in CCl_4_-treated mice receiving PBS (n = 6) or the different recombinant proteins IFNγ (n = 6), MimIFNγ (n = 5), BiPPB-IFNγ (n = 6), BiPPB-mimIFNγ (n = 6). Bars represent mean ± SEM of 5–6 mice per group. The results showed a significant reduction in platelet counts following two intravenous administration of IFNγ, which was significantly improved following treatment with BiPPB-mimIFNγ and to a lesser extent with BiPPB-IFNγ. MimIFNγ did not show significant change in platelets counts due to lack of binding to IFNγR or PDGFβR. *P<0.05, **P<0.01 denotes significance versus PBS treated CCl_4_ mice. #P<0.05 denotes the significance versus IFNγ-treated CCl_4_ mice.

The main hurdles in IFNγ-based therapies are the adverse effects that led to the failure of clinical trials. IFN-mediated reduction in blood platelets is clinically relevant and a well-known side effect [30]. Indeed, we observed a significant reduction in the platelet counts following two intravenous treatments with IFNγ (p<0.001), which was significantly improved following treatment with BiPPB-mimIFNγ and to a lesser extent with BiPPB-IFNγ (**Figure 6**). MimIFNγ did not induce a significant change in platelets counts due to lack of binding to IFNγR or PDGFβR.

## Discussion

Hepatocellular damage, hepatic inflammatory cell infiltration and extensive tissue remodeling ultimately culminate into the development of progressive fibrosis and cirrhosis [31]. During liver fibrosis, the interplay between growth factors and cytokines produced by the damaged hepatocytes; inflammatory cells and non-parenchymal cells activate quiescent hepatic stellate cells (HSC). These activated HSC in turn proliferate and accumulate in the injured liver producing large amounts of extracellular matrix proteins [32]. Therefore, therapeutic approaches to silence these activated HSC would be appropriate to inhibit or reverse liver fibrosis [12].

Fibrosis is an heterogeneous process e.g. CCl_4_ intoxication results in hepatocyte damage, necrosis, inflammation, and fibrosis, leading to portal fibrosis or cirrhosis. While bile duct ligation (BDL) stimulates the proliferation of biliary epithelial cells and oval cells, resulting in proliferating bile ductules accompanied by portal inflammation leading to biliary cirrhosis (PBC). As documented in fibrotic animal models (BDL or CCL_4_ etc.) and diseased human livers, PDGFβR is expressed abundantly on activated HSC or portal fibroblasts (collectively referred as myofibroblasts-like cells) [21], [22]. The expression was found to be very weak in normal healthy tissues or cells (pericytes or smooth muscle cells) relative to expression levels in the fibrotic livers, which motivated us to examine the potential of using PDGFβR for a targeted delivery of anti-fibrotic or apoptotic drugs to the fibrotic livers [12]. In the present study, we have directed IFNγ or mimetic IFNγ (signaling moiety of IFNγ lacking extracellular IFNγR binding site while retaining activities of IFNγ) [33] to PDGFβR-expressing activated HSC using bicyclic PDGFβR peptides to inhibit liver fibrosis with reduced adverse effects.

IFNγ or mimetic IFNγ modified with bicyclic PDGFβR-recognizing peptide were generated via recombinant technology in *E.coli*. Unmodified IFNγ or mimetic IFNγ were also produced in parallel to serve as respective controls. BiPPB-IFNγ and BiPPB-mimIFNγ were produced from a periplasmic vector (pET39b), since this vector contains the DsbA tag that exports the expressed proteins to the periplasmic space (space between plasma membrane and outer membrane). DsbA, periplasmic enzyme catalyzes the sequential formation of disulfide bonds therefore favors proper folding of the fusion proteins containing bicyclic peptide (cyclized via bisulfide bonds) at the N-terminus of IFNγ and mimetic IFNγ. Unmodified IFNγ and mimetic IFNγ were produced using pET42a vector as cytoplasmic proteins. These proteins were expressed, purified and analyzed using immune dot-blots where the presence of IFNγ, mimIFNγ, BiPPB-IFNγ and BiPPB-mimIFNγ was confirmed using anti-IFNγ and anti-PPB antibodies.

The BiPPB-modified proteins were investigated for PDGFβR-specific binding in human HSC. BiPPB-IFNγ and BiPPB-mimIFNγ showed specific binding to PDGFβR-expressing human LX2 cells whereas unmodified mouse derived IFNγ and mimIFNγ did not show binding to these human cells due to species restrictive interaction of mouse IFNγ to mouse IFNγR, while PDGFβR interaction is not species-specific. BiPPB-IFNγ and BiPPB-mimIFNγ also induced a strong reduction in TGFβ-induced collagen I expression in human LX2 cells while IFNγ and mimIFNγ did not affect the collagen expression in these cells corroborating with the binding studies. We did not observe any difference in α-SMA expression *in vitro* after 48 hrs of treatment (data not shown) while significant reduction is observed in collagen expression (**Figure 3A, 3B**), since IFNγ has shown to have direct effect on collagen expression by directly acting on C/EBPbeta signaling pathway [34] while longer treatments might lead to inhibitory effects on α-SMA expression. These studies clearly demonstrate that directing IFNγ or mimetic IFNγ to the accessory PDGFβR can transform mouse (whose activity is restricted to mouse cells) and mim-IFNγ (unable to enter any cell), into highly active proteins in human LX2 cells. On the other hand, we studied effect of these recombinant proteins in mouse macrophages expressing IFNγR and lacking PDGFβR. Results showed that IFNγ or BiPPB-modified IFNγ containing IFNγR binding site activated mouse RAW cells and induced NO release while both mimIFNγ (lacking IFNγR binding site) or BiPPB-modified mimIFNγ (containing PDGFβR binding site and lacking IFNγR binding site) did not show any effect in macrophages. These results indicate that targeted mimIFNγ will not influence cell types (especially macrophages which are known to be highly influenced by IFNγ) other than PDGFβR-expressing cells and therefore will not induce adverse effects in other normal cells or tissues.

In the past years, our group has extensively demonstrated the higher liver accumulation and HSC-specific distribution of PPB-modified proteins [35]. Here we examined the intra-hepatic activation of IFNγ signaling pathway *in vivo* in treated livers to assess the increased accumulation of our targeted proteins in fibrotic livers. IFNγ internalization results in the activation of the JAK–STAT pathway and subsequent phosphorylation of signal transducers and activators of transcription (STAT1) that binds to unique gamma-activated sequence (GAS) regulating IFNγ-responsive genes [36]. Both BiPPB-modified IFNγ and mimIFNγ induced a significant increase in intrahepatic pSTAT1α signalling compared to unmodified IFNγ and mimiFNγ implicating increased liver accumulation of targeted proteins.

Thereafter, we studied the anti-fibrotic effects of recombinant proteins in the CCl_4_-induced liver fibrogenesis model in mice. This model is associated with HSC activation, enhanced PDGFR expression and ECM deposition, the key parameters of fibrogenesis. Two subsequent intravenous injections of IFNγ, BiPPB-IFNγ or BiPPB-mimIFNγ led to the highly significant reduction in collagen I expression (major extracellular matrix protein) and α-SMA (HSC activation marker) expression. Additionally, IFNγ, BiPPB-IFNγ and BiPPB-mimIFNγ enhanced the MMP13/TIMP1 transcripts ratio implying activation of fibrolysis. In this study, we observed anti-fibrotic effects with IFNγ in comparison with our previously reported studies [20], [23], is attributed to the increased dose (5 µg) used here, as compared to 2.5 µg dose used earlier. MimIFNγ that cannot be internalized due to lack of receptor binding sites did not show any effect on these fibrotic PPB-modified proteins can block the PDGFβR in vitro [35], [37] and this might also account for the observed antifibrotic effects. However, previous studies with PPB-peptides coupled to albumin have shown that this effect does not occur *in vivo* at the doses used [20], [23]. To further reinforce this hypothesis, we used synthetic BiPPB as a control but did not observed any effect on the fibrotic parameters (data not shown).

Liver fibrosis or cirrhosis is a slowly progressing disease that develops over many years, therefore patients are treated for longer periods to cure or reverse the disease. Therefore, therapies with improved therapeutic efficacy and preferably without provoking off-target systemic effects would be highly favourable. In clinical trials with IFNγ, patients suffered from mild to severe adverse effects; therefore we investigated one of the well-known IFN-mediated adverse effects, significant reduction in circulating blood platelets (mainly produced by bone marrow) can lead to fatal disorders and also found to be associated with the serotonin levels associated to depression [30]. We found that only two intravenous injections of IFNγ led to a highly significant reduction in platelet counts (p<0.001) in comparison to PBS-treated mice. Furthermore, BiPPB-IFNγ that can still interact with IFNγR receptor showed a slight reduction in platelet counts (p<0.05), but mimIFNγ (without receptor binding sites) and BiPPB-mimIFNγ (only specific to PDGFβR) did not induce a reduction in platelet counts. Further studies in long-term fibrotic models are important to examine the adverse effects of targeted constructs due to the long-term administration. Since platelets are produced by bone marrow, it is also important to study the effect of these constructs on the bone marrow as these effects may influence other crucial biological processes.

In conclusion, the results in this paper clearly demonstrate that HSC-targeted mimIFNγ is capable of accumulating in PDGFβR-expressing fibrotic livers and exerting an inhibitory effect on fibrotic parameters, which makes this recombinant protein a highly attractive candidate to explore for the treatment of liver fibrosis. The recombinant synthesis of this chimeric compound will facilitate further translational research with this compound.

## Supporting Information

Figure S1 **Schematic representation depicting cloning strategy for preparation of pET42a-IFNγ.** PCR amplified and EcoRI digested mouse IFNγ gene fragment was cloned in pET42a (+) prokaryotic vector at Psh A1/Eco RI site.
(TIF)

Figure S2 **Schematic representation depicting cloning strategy for preparation of pET42a-mimIFNγ.** PCR amplified and EcoRI digested mouse mimetic IFNγ gene fragment was cloned in pET42a (+) prokaryotic vector at Psh A1/Eco RI site.
(TIF)

Figure S3 **Schematic representation depicting cloning strategy for preparation of pET39b-BiPPB.** BiPPB was prepared by annealing and PCR extension of 2 sets of primers (refer to methods), which were linked together via Bam HI site and cloned in Sca I/Not I digested pET39b (+) prokaryotic vector.
(TIF)

Figure S4 **Schematic representation depicting cloning strategy for preparation of pET39b-BiPPB-IFNγ.** PCR amplified IFNγ prepared for fusion with BiPPB was digested with Not I/Xho I was cloned in pET39b vector at Not I/Xho I site. The resultant recombinant vector termed as pET39b-BiPPB-IFNγ encodes for BiPPB and IFNγ plus a three amino-acid linker (AAA) between them.
(TIF)

Figure S5 **Schematic representation depicting cloning strategy for preparation of pET39b-BiPPB-mimIFNγ.** PCR amplified mimIFNγ prepared for fusion with BiPPB was digested with Not I/Xho I was cloned in pET39b vector at Not I/Xho I site. The resultant recombinant vector termed as pET39b-BiPPB-mimIFNγ encodes for BiPPB and mimIFNγ plus a three amino-acid linker (AAA) between them.
(TIF)

Figure S6 ***In vivo*** **activation of IFNγ pSTAT1α signaling pathway by recombinant proteins.** Representative western blot images of pSTAT1α (upper panel) and β-actin (house-keeping protein, lower panel) after 24 h post intravenous administration of IFNγ (n = 6), MimIFNγ (n = 5), BiPPB-IFNγ (n = 6), BiPPB-mimIFNγ (n = 6) or PBS alone (n = 6). Figure shows the significant increase in activation of pSTAT1α with HSC-targeted fusion proteins (BiPPB-IFNγ, BiPPB-mimIFNγ).
(TIF)

Figure S7 **Effects of recombinant proteins on fibrotic parameters in vivo.** Representative western blot images of collagen I (upper panel) and β-actin (house-keeping protein, lower panel) from CCl_4_-treated mice (acute model) that were treated with IFNγ (n = 6), MimIFNγ (n = 5), BiPPB-IFNγ (n = 6), BiPPB-mimIFNγ (n = 6) or PBS alone (n = 6). Figure shows the significant reduction in collagen expression after treatment with HSC-targeted fusion proteins (BiPPB-IFNγ, BiPPB-mimIFNγ).
(TIF)
