# Supplementary figures and images for: Targeted Recombinant Fusion Proteins of IFNγ and Mimetic IFNγ with PDGFβR Bicyclic Peptide Inhibits Liver Fibrogenesis In Vivo
Source: PLoS One. 2014 Feb 24;9(2):e89878. doi: 10.1371/journal.pone.0089878 (PMC3933682; doi:10.1371/journal.pone.0089878)

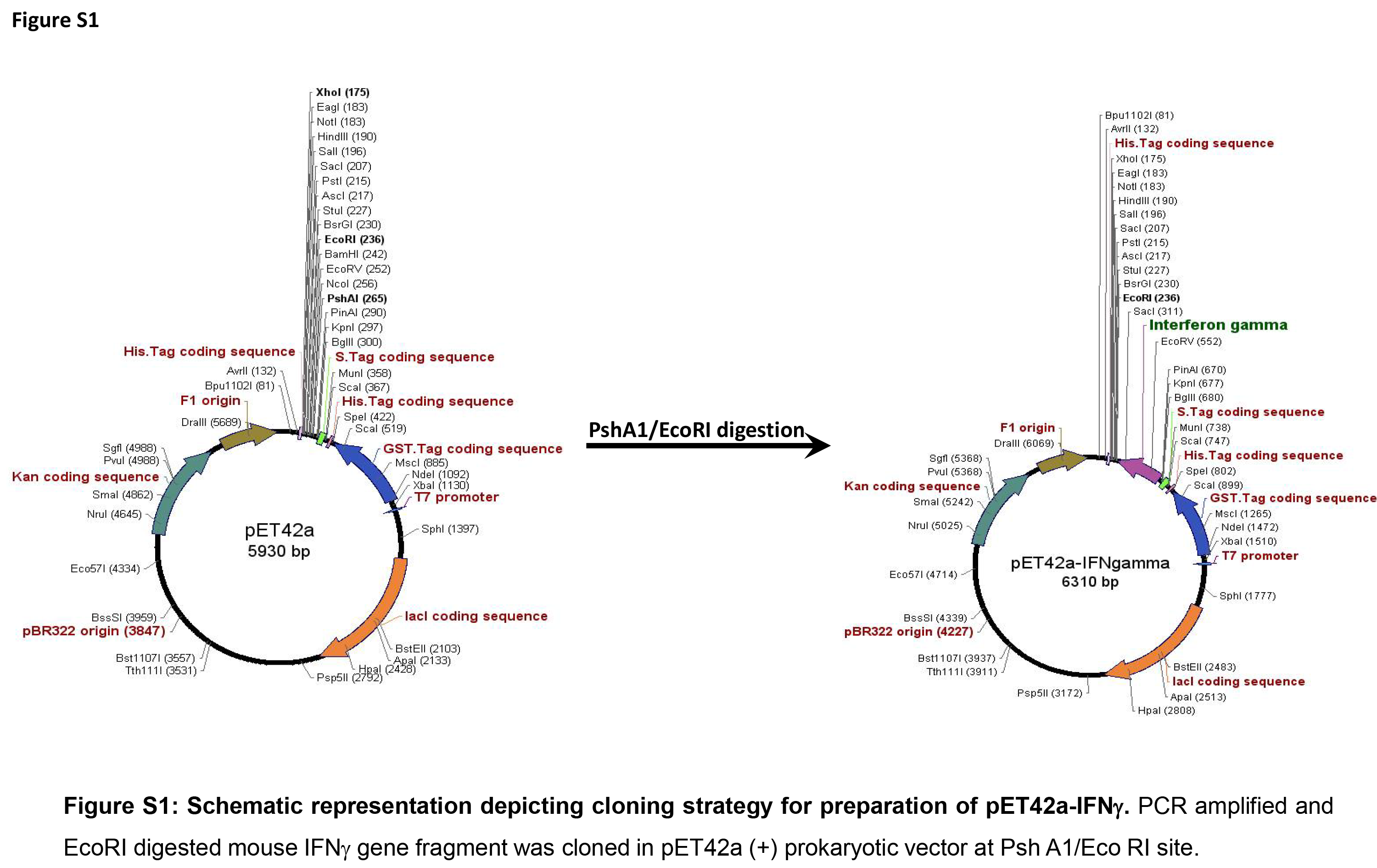

Supplement: Figure S1 — Schematic representation depicting cloning strategy for preparation of pET42a-IFNγ. PCR amplified and EcoRI digested mouse IFNγ gene fragment was cloned in pET42a (+) prokaryotic vector at Psh A1/Eco RI site. (TIF) [file pone.0089878.s001.tif]

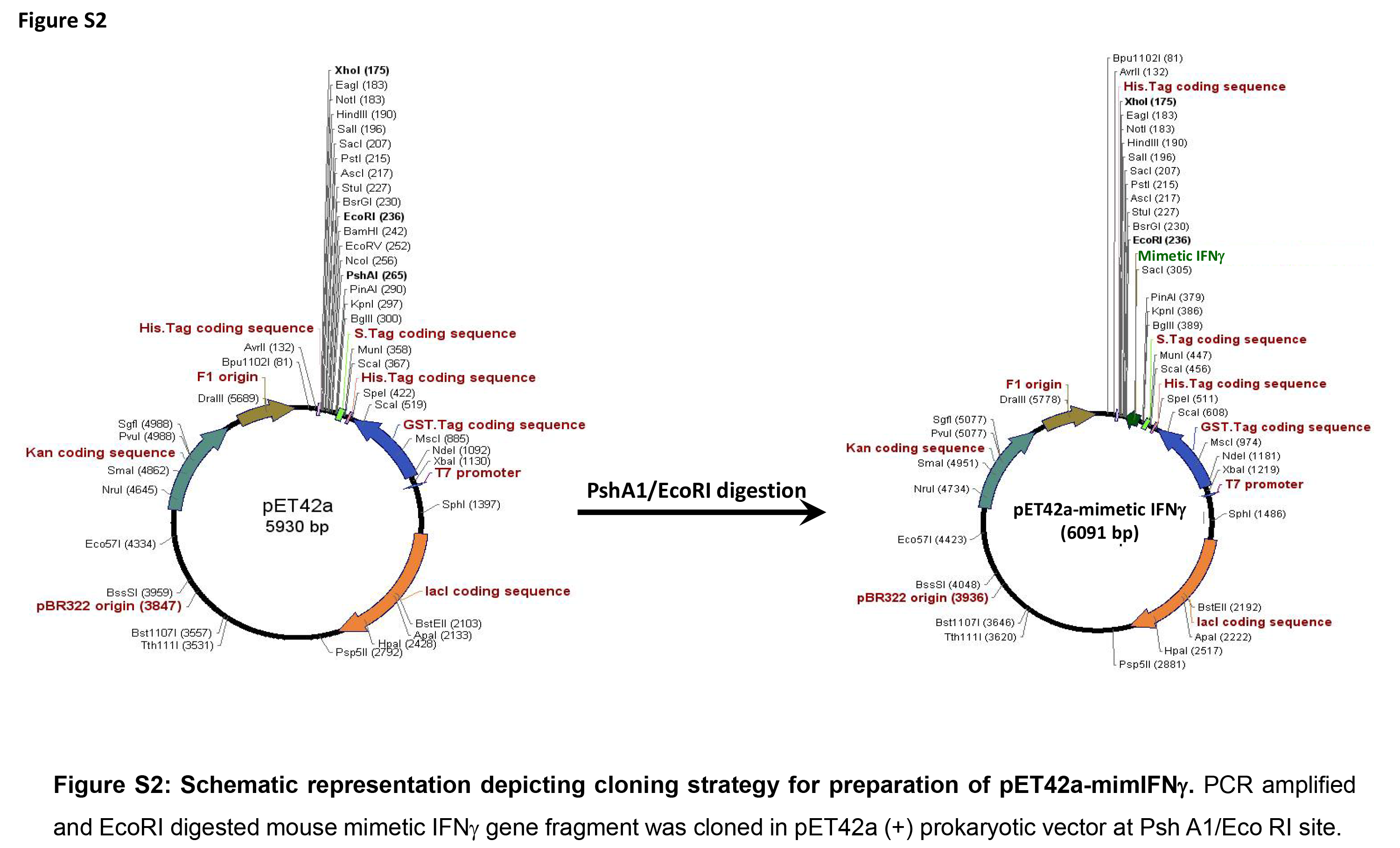

Supplement: Figure S2 — Schematic representation depicting cloning strategy for preparation of pET42a-mimIFNγ. PCR amplified and EcoRI digested mouse mimetic IFNγ gene fragment was cloned in pET42a (+) prokaryotic vector at Psh A1/Eco RI site. (TIF) [file pone.0089878.s002.tif]

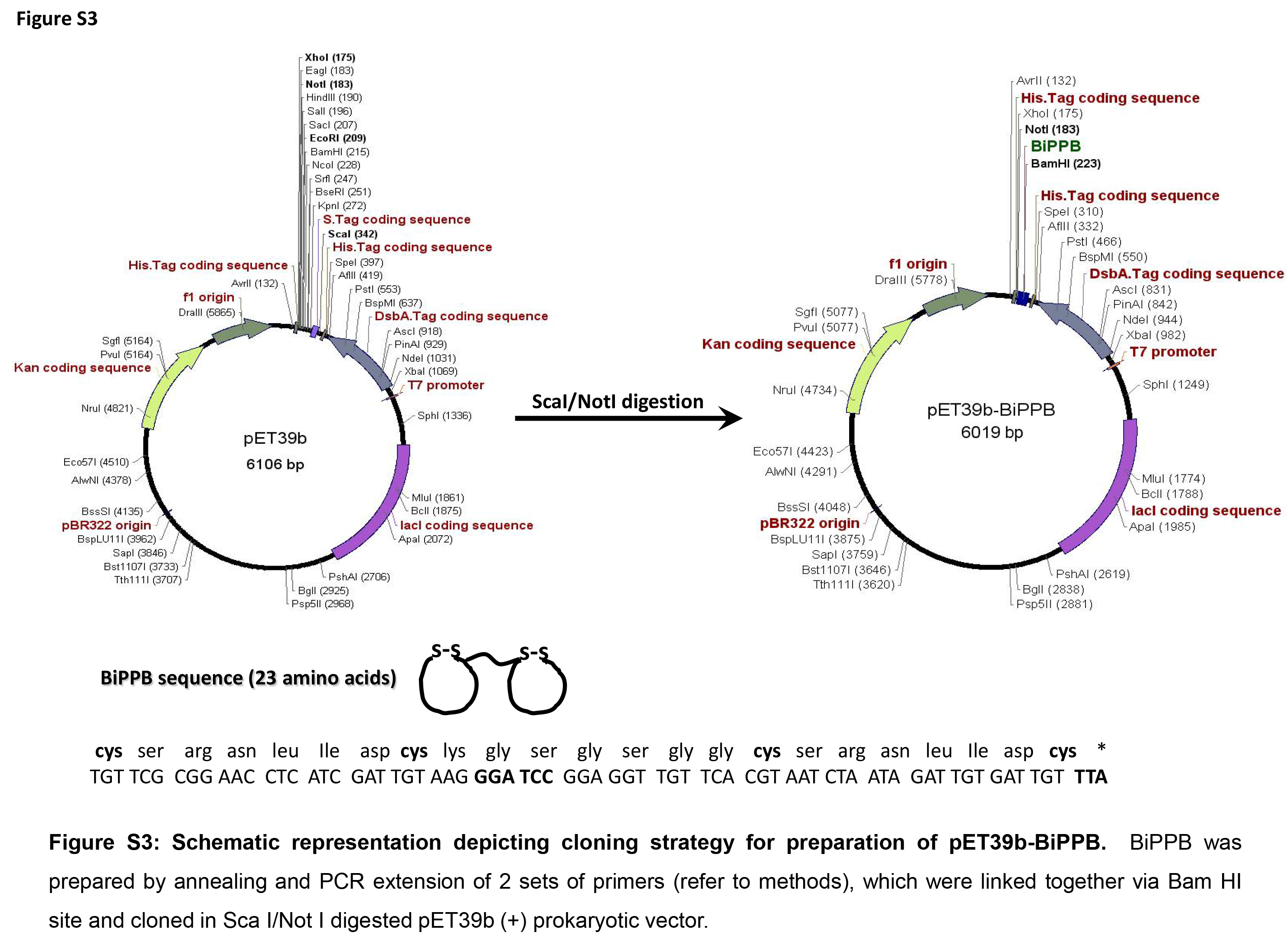

Supplement: Figure S3 — Schematic representation depicting cloning strategy for preparation of pET39b-BiPPB. BiPPB was prepared by annealing and PCR extension of 2 sets of primers (refer to methods), which were linked together via Bam HI site and cloned in Sca I/Not I digested pET39b (+) prokaryotic vector. (TIF) [file pone.0089878.s003.tif]

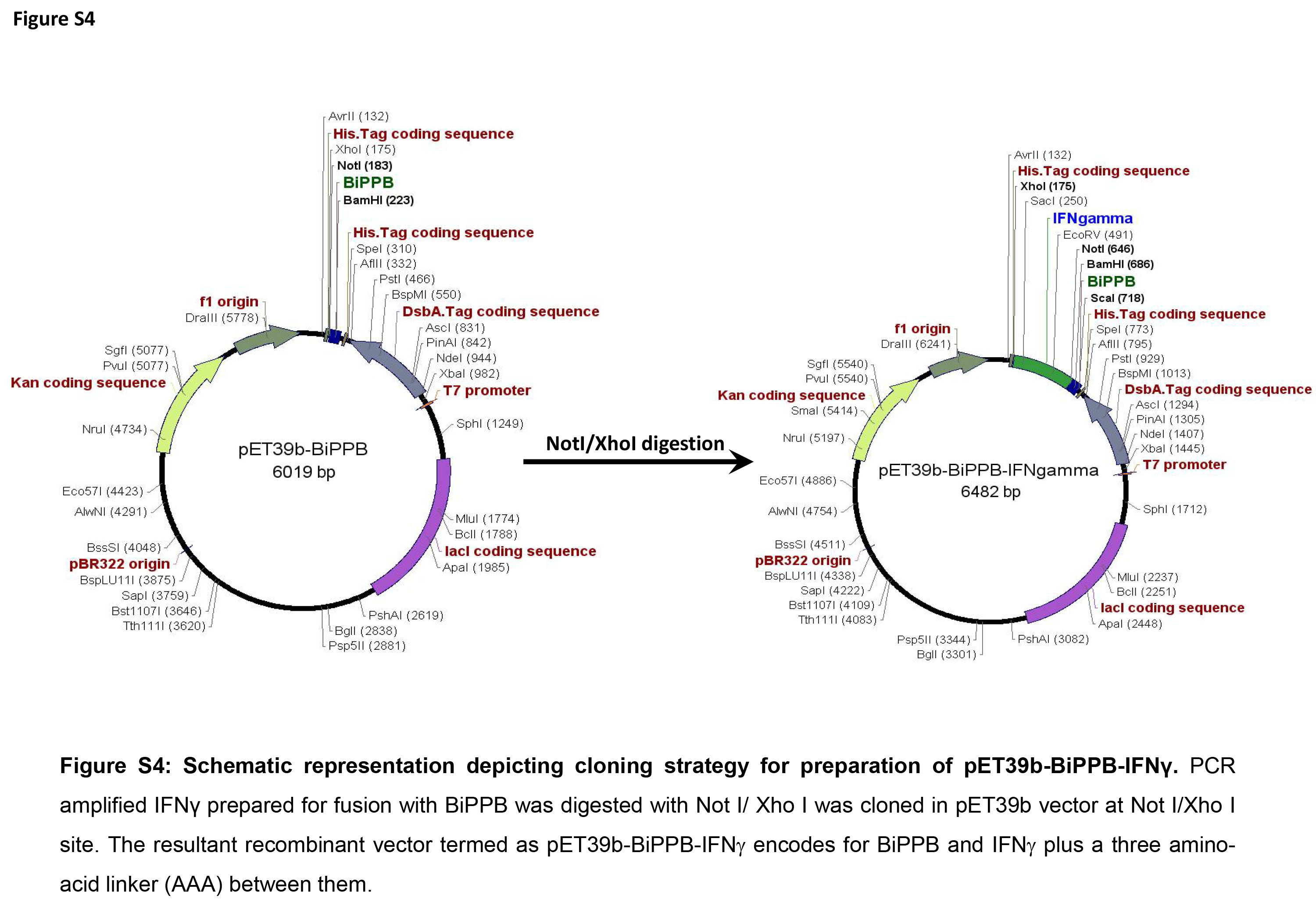

Supplement: Figure S4 — Schematic representation depicting cloning strategy for preparation of pET39b-BiPPB-IFNγ. PCR amplified IFNγ prepared for fusion with BiPPB was digested with Not I/Xho I was cloned in pET39b vector at Not I/Xho I site. The resultant recombinant vector termed as pET39b-BiPPB-IFNγ encodes for BiPPB and IFNγ plus a three amino-acid linker (AAA) between them. (TIF) [file pone.0089878.s004.tif]

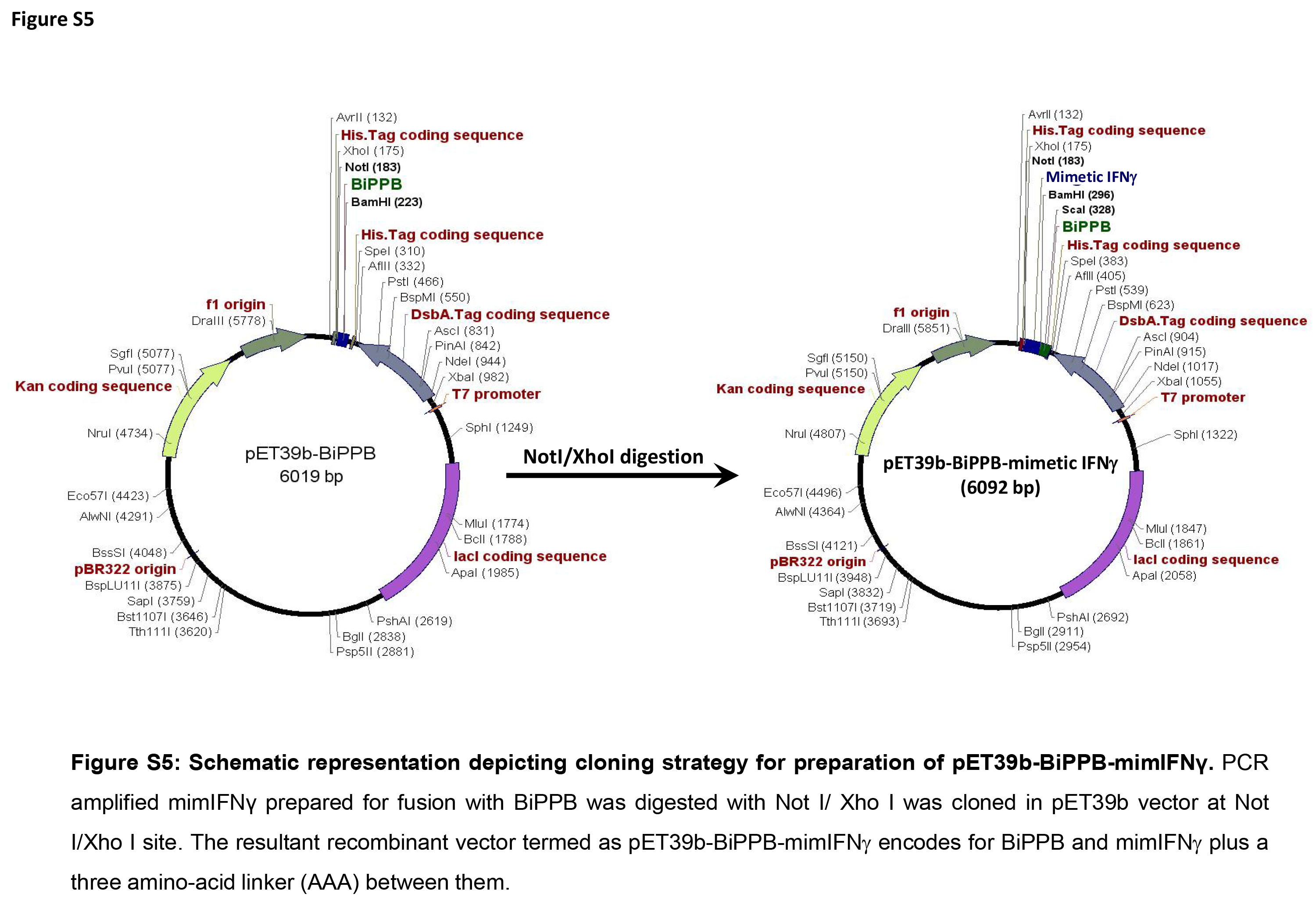

Supplement: Figure S5 — Schematic representation depicting cloning strategy for preparation of pET39b-BiPPB-mimIFNγ. PCR amplified mimIFNγ prepared for fusion with BiPPB was digested with Not I/Xho I was cloned in pET39b vector at Not I/Xho I site. The resultant recombinant vector termed as pET39b-BiPPB-mimIFNγ encodes for BiPPB and mimIFNγ plus a three amino-acid linker (AAA) between them. (TIF) [file pone.0089878.s005.tif]

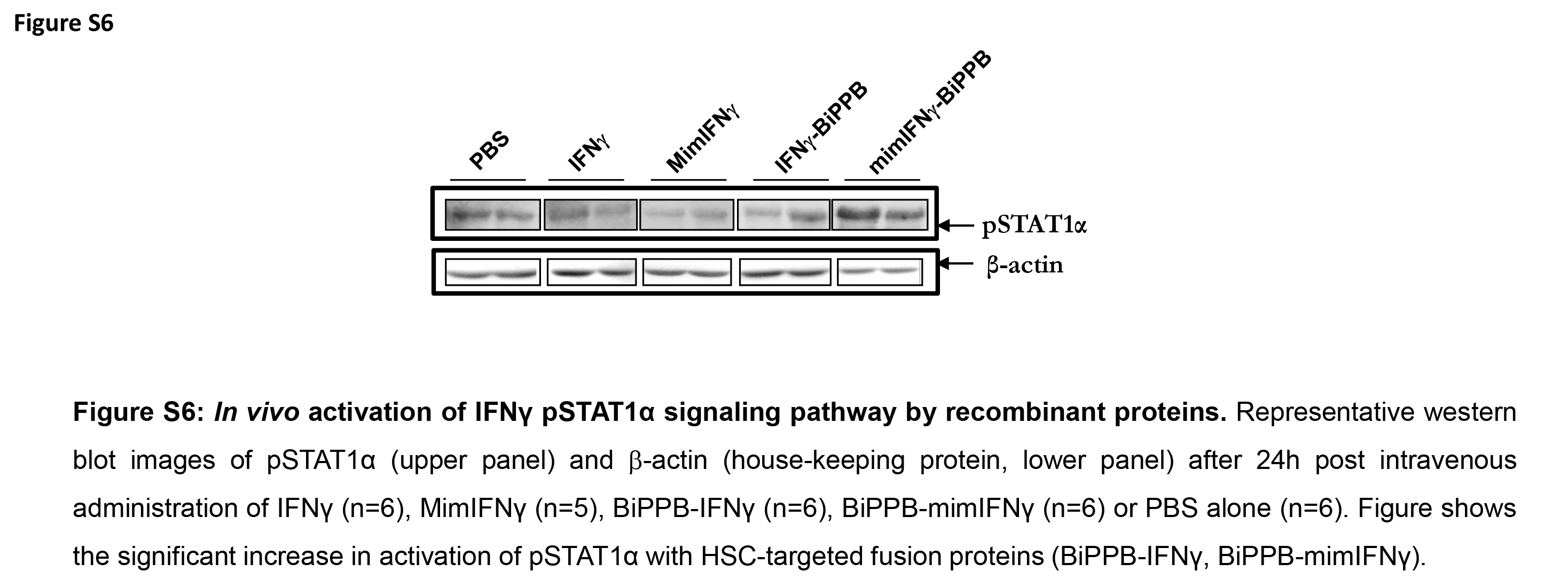

Supplement: Figure S6 — In vivo activation of IFNγ pSTAT1α signaling pathway by recombinant proteins. Representative western blot images of pSTAT1α (upper panel) and β-actin (house-keeping protein, lower panel) after 24 h post intravenous administration of IFNγ (n = 6), MimIFNγ (n = 5), BiPPB-IFNγ (n = 6), BiPPB-mimIFNγ (n = 6) or PBS alone (n = 6). Figure shows the significant increase in activation of pSTAT1α with HSC-targeted fusion proteins (BiPPB-IFNγ, BiPPB-mimIFNγ). (TIF) [file pone.0089878.s006.tif]

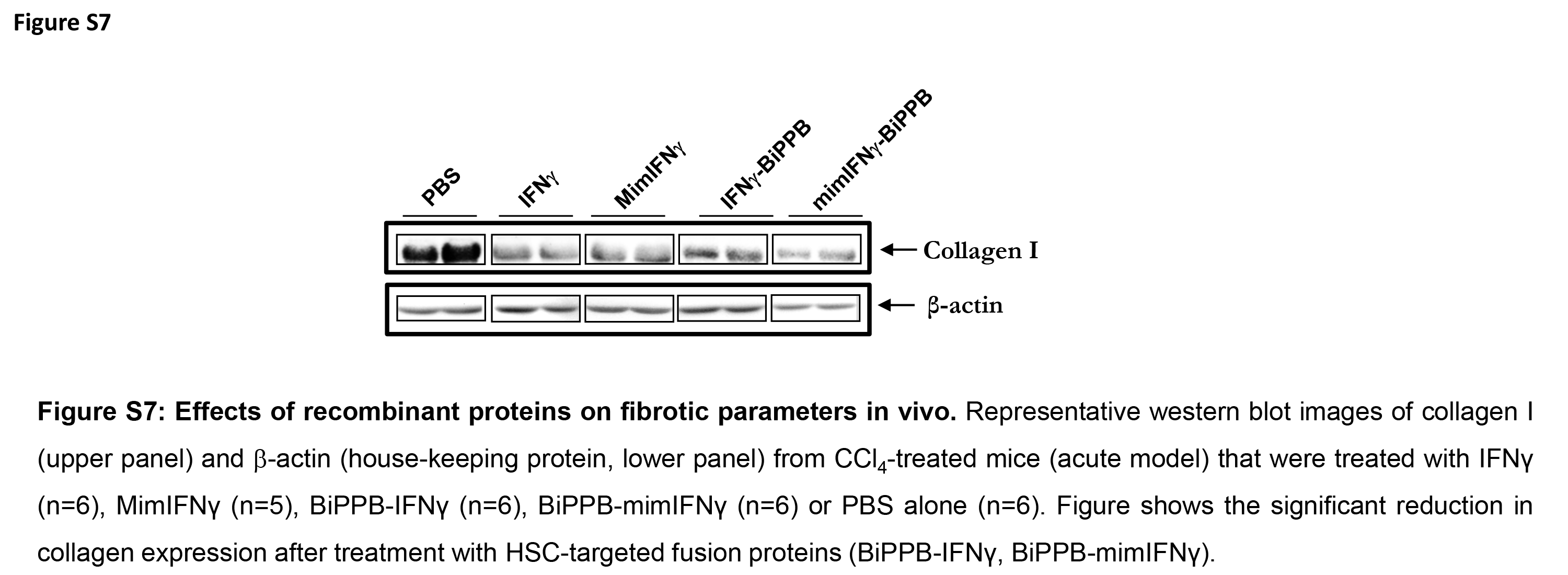

Supplement: Figure S7 — Effects of recombinant proteins on fibrotic parameters in vivo. Representative western blot images of collagen I (upper panel) and β-actin (house-keeping protein, lower panel) from CCl4-treated mice (acute model) that were treated with IFNγ (n = 6), MimIFNγ (n = 5), BiPPB-IFNγ (n = 6), BiPPB-mimIFNγ (n = 6) or PBS alone (n = 6). Figure shows the significant reduction in collagen expression after treatment with HSC-targeted fusion proteins (BiPPB-IFNγ, BiPPB-mimIFNγ). (TIF) [file pone.0089878.s007.tif]
